# ‎ Recurrence in Patients with Bipolar Disorder and Its Risk Factors

**Published:** 2016-07

**Authors:** Roya Najafi-Vosough, Ali Ghaleiha, Javad Faradmal‎, Hossein Mahjub

**Affiliations:** 1 Department of Biostatistics, School of Public Health, Hamadan University of Medical ‎Sciences, Hamadan, Iran. Email: n_vosough@yahoo.com; 2 Behavioral Disorders and Substance Abuse Research Center, Hamadan University of Medical ‎Sciences, Hamadan, Iran. Email: ‎dralighaleiha@gmail.com; 3 Modeling of Noncommunicable Diseases Research Center, Hamadan University of Medical Sciences, Hamadan, Iran. Email: javad.faradmal@umsha.ac.ir ‎; 4 Research Center for Health Sciences, Hamadan University of Medical Sciences, Hamadan, Iran.

**Keywords:** *Bipolar Disorder*, *Ordinal Logistic Regression*, *Prognosis Factors*, *Recurrence*

## Abstract

**Objective: **The aim of this study was to identify prognosis factors associated with recurrence in patients ‎with bipolar disorder.‎‎

**Method: **This retrospective cohort study was conducted in Hamadan Province, the west of Iran. All ‎patients (n = 400) with bipolar disorder who were hospitalized for the second time or more ‎during April 2008 to September 2014 were included in this study. Ordinal logistic regression ‎analysis was employed to determine the effective factors in each recurrence, and odds ratio (OR) and 95% confidence intervals (CI) were obtained.‎

**Results: **The mean (SD) age of the participants at the entrance to the study was 34.62 (11.68) years. ‎There was an association between recurrence and type of bipolar disorder (P = 0.033). The ‎OR of recurrence was 0.28 (95% CI: 0.09, 0.90) for bipolar disorder II; 0.35 (95% CI: 0.13, ‎‎0.92) for the patients‎‏ ‏with college education; 0.39 (95% CI: 0.25, 0.60) for employed ‎patients; 0.55 (95% CI: 0.35, 0.87) for patients who received both drugs and ‎electroconvulsive therapy, and 1.89 (95% CI: 1.23, 2.92) for patients who stopped using ‎drugs. In addition, a non-significant association was found between recurrence and age, sex, ‎marital status, place of residence, season, mood classification and family history of mood ‎disorder.‎

**Conclusion: **Type of bipolar disorder and cessation of medication were the leading causes of an increase in ‎the relapse of the disease. Furthermore, patients who received both drugs and ‎electroconvulsive therapy had a fewer risk of recurrence.‎

Bipolar disorder is defined as a severe, chronic, disabling mental disorder with significant ‎effects on the economy, the patient and the society (1, 2). According to the fourth edition of ‎Diagnostic and Statistical Manual of Mental Disorders Text Revision (DSM-IV-TR), two ‎major categories of bipolar disorder exist: Bipolar I disorder and bipolar II disorder (3). ‎Bipolar disorders types I and II affect about 2% of the world’s population (4). The lifetime ‎prevalence of this illness was reported differently in different studies. The reported lifetime ‎prevalence of bipolar disorder I is between 0 and 2.4%, and for the whole bipolar spectrum, in ‎which bipolar disorder II and cyclothymia and hypomania are also included, it is between 2.6 ‎and 7.8%. The annual incidence of bipolar illness is less than one percent (5). ‎

Gaining knowledge about the course of bipolar disorder is of prime importance. Bipolar ‎disorders have long episodes, and many patients experience recurrence during their lifetimes. ‎Close to 60% of the patients experience a recurrence of bipolar disorder in the first two years, ‎and about 75% experience a recurrence in over five years following the initial diagnosis (6,7). ‎Salvatore et al (1) indicated that the risks of relapse and recurrence were very high in the first ‎two years. ‎World Health Organization reported that bipolar disorder is the sixth leading cause of life ‎long disability among persons aged 15 to 44 years worldwide (8). In some studies, it was ‎reported that non-adherence to treatment and family history of mood disorder are associated ‎with an increased number of episodes and more hospitalizations (9-11). Studies have shown ‎that a poor occupational status, alcohol dependence and male gender were all factors that ‎contributed to a poor prognosis (5). ‎

Joy Albuquerque et al. (12) investigated the recurrence rates in Ontario physicians monitored ‎for major depression and bipolar disorder. They made exploratory analysis on recurrence ‎predictors including age, sex, psychiatric diagnosis, psychiatric comorbidity, medical ‎comorbidity, number of past episodes, past hospitalizations, and family history of psychiatric ‎disorder. They concluded that recurrence rates are high and markedly hastened by the ‎presence of psychiatric comorbidity.‎ in the world and especially in Iran; few studies have been done to find the possible ‎relationships between prognostic factors and recurrence of bipolar disorders. Since preventing ‎recurrence in the patients is important, the aim of this study was to identify the prognosis ‎factors of recurrence among patients with bipolar disorder.‎ ‎

## Materials and Method

This retrospective cohort study was conducted in Hamadan Province, the west of Iran, from ‎April 2008 to September 2014. The population of this study was patients with bipolar ‎disorder with more than one recurrence leading to hospitalization in Farshchian hospital, the ‎only psychiatric hospital in Hamadan province. Data were extracted from hospital records ‎using a checklist of items according to the context of the patients' records and clinical ‎examination of psychiatrists.‎

The checklist included data on demographic variables such as age, sex, marital status, ‎education, occupation, place of residence and ethnicity. Moreover, information on the disease ‎such as type of treatment, season, mood classification, family history of mood disorder, and ‎cessation of medication were assessed. Recurrence was the response variable.‎

Ordinal logistic regression analysis was performed to assess the effect of various risk factors ‎on recurrence. Odds ratio (OR) was reported to address the association between recurrence ‎and the associated predictors. All statistical analyses were performed at a significance level of ‎‎0.05 using Stata software, Version 11 (Stata Corp, College Station, TX, USA).‎

## Results

All the patients with bipolar disorder (n = 400) who were hospitalized for the second time or ‎more during April 2008 to September 2014 were included in this study. 

**Table1:** Characteristics of the Patients with Bipolar Disorder

** Variables**	**Number**	**Percent**
** Sex**		
Male	288	72.0
Female	112	28.0
** Marital status**		
Married	189	47.3
Single	161	40.3
Divorced/ Widowed	50	12.5
** Education**		
Illiterate	39	10.1
Without College education	304	79.0
College education	42	10.9
** Occupation**		
Employed	207	51.8
Unemployed	193	48.3
** Region**		
Urban	272	68.3
Rural	126	31.7
** Ethnicity**		
Persian	176	44.0
Other †	224	56.0
** DSM-IV-TR classification**		
Bipolar I disorder	389	97.3
Bipolar II disorder	11	2.8
** Season**		
Spring	89	22.3
Summer	115	28.8
Autumn	106	26.5
Winter	90	22.5
** Type of treatments**		
Drug	268	67
Drug and ECT ‡	116	29
Drug and Psychosocial treatments	16	4
** Mood classification**		
Manic episode	256	69.9
Depressed episode	96	26.2
Mixed episode	14	3.8
** Family history of mood disorder ** §		
No	280	70
Yes	120	30
** Cessation of medication**		
No	244	61
Yes	156	39
** Number of recurrence**		
2	150	37.5
3	126	31.5
4	76	19.0
5	27	6.8
6	14	3.5
7	7	1.8

† Turkish, Lurish or Kurdish.

‡ Electroconvulsive therapy.

§ Family history with mood disorder included of Bipolar or Unipolar disorder

**Table2:** The Odds Ratio (OR) of Different Variable on Reccunce of Patients with Bipolar Disorder

**Variables**	**OR (95% CI)**	**P value**
** Age (yr.)**	1.01(0.99, 1.04)	0.138
** Sex**		
Male	1.00	
Female	0.71(0.44, 1.13)	0.150
** Marital status**		
Married	1.00	
Single	0.96(0.54, 1.70)	0.890
Divorced/ Widowed	1.00(0.50, 1.88)	0.945
** Education**		
Illiterate	1.00	
Without College education	0.79(0.37, 1.67)	0.545
College education	0.35(0.13, 0.92)	0.035
** Occupation**		
Unemployed	1.00	
Employed	0.39(0.25, 0.60)	0.001
** Region**		
Urban	1.00	
Rural	0.73(0.46, 1.16)	0.195
** Ethnicity**		
Persian	1.00	
Other †	0.56(0.37, 0.87)	0.009
** DSM-IV-TR classification**		
Bipolar I disorder	1.00	
Bipolar II disorder	0.28(0.09, 0.91)	0.033
** Season**		
Spring	1.00	
Summer	1.09(0.62, 1.92)	0.755
Autumn	1.13(0.63, 2.04)	0.670
Winter	0.69(0.37, 1.29)	0.253
** Type of treatments**		
Drug	1.00	
Drug and ECT ‡	0.55(0.35, 0.87)	0.011
Drug and Psychosocial treatments	2.82(0.97, 8.17)	0.056
** Mood classification**		
Manic episode	1.00	
Depressed episode	1.20(0.75, 1.91)	0.437
Mixed episode	1.25(0.45, 3.51)	0.659
** Family history of mood disorder ** §		
No	1.00	
Yes	1.42(0.91, 2.20)	0.116
** Cessation of medication**		
No	1.00	
Yes	1.89(1.23, 2.92)	0.004

† Turkish, Lurish or Kurdish

‡ Electroconvulsive therapy

§ Family History with Mood Disorder including Bipolar or Unipolar Disorder

The mean (SD) age ‎of the participants at the time they entered into the study was 34.62(11.68) years. The number ‎of male patients was 288 (72%) and the number of females was 112 (28%); the number of ‎patients who lived in urban and rural areas was 272 (68%) and 126 (31%), respectively. The ‎minimum and maximum recurrence observed during the study period was 2 and 7, ‎respectively. The characteristics of patients are given in Table 1.‎

The results of proportional ordinal logistic regression analysis are presented in Table 2. An ‎increase in relapse rate was observed with an increase in age. A non-significant association ‎was detected between recurrence and the group with no college education compared to ‎illiterate patients. However, a significant association was detected between the group with ‎college education and recurrence (P = 0.035). Moreover, a strong association was found ‎between occupation and recurrence, and the OR of recurrence in employed patients was 0.39 ‎‎(95% CI: 0.25, 0.60) compared to the unemployed. The recurrence in non-Persian (Kurdish, ‎Turkic and Lurish) patients was significant compared to the Persian (P = 0.009). ‎

There was a significant association between bipolar II disorder and recurrence compared to ‎bipolar I disorder (P = 0.033). ‎

The cessation of medication was a strong risk factor in recurrence of the disease. The OR of ‎recurrence in patients who ceased the use of drugs was 1.89 (95% CI: 1.23, 2.92) compared ‎to those who used drugs. Compared to patients with manic episode, the patients with ‎depressed or mixed episode were at higher risk of recurrence. However, these associations ‎were not statistically significant. Treatment with either drugs or electroconvulsive therapy, ‎alone had an association with recurrence (P = 0.011). In addition, when treatment, drugs and ‎ECT were administered together, they decreased the risk of recurrence.‎

## Discussion

The results of this study revealed that recurrence was associated with various predictors ‎including education, occupation, ethnicity, and type of bipolar disorder according to DSM-‎IV-TR, type of treatment and cessation of medication.‎

With increasing age there was an increasing risk of recurrence and this result confirmed the ‎study done in 2006 (13); however, no significant effect was detected for age. In some ‎studies, the reported percentage of hospitalization of men was more than that of women, ‎urban was more than rural and married was more than single or Divorced/ Widowed‏ ‏‎(14,15). ‎The results of this study confirmed that the percentage of men who were hospitalized was ‎more than that of women. No significant association was found between recurrence and sex, ‎which correspond with the study done by Joy Albuquerque et al. (12). In 2003, Kupka et al. ‎‎ (16) found that the risk of bipolar disorder in women is significantly higher compared to men. ‎Although no significant effects of sex and place of residence were found, the risk of ‎recurrence among men was more compared to women, and it was more in the urban than in ‎the rural areas. In 2010, Peen et al. (17) indicated a greater prevalence of mood disorders in ‎urban areas compared to the rural. We found that the risk of recurrence was the same in ‎divorced, widowed and married patients.‎

‎In a study conducted by Perlis et al. (13), no significant effect was detected between number ‎of recurrence and education. The results of this study revealed that the risk of recurrence ‎decreased in patients with college education. No significant effect was observed for season in ‎patients in this study, but the risk of recurrence was increased in the autumn and summer. ‎

We found a strong association between occupation status and recurrence. Dickerson et al. ‎‎ (18) investigated the association between cognitive functioning and employment status of ‎persons with bipolar disorder and concluded that current employment status was significantly ‎associated with history of psychiatric hospitalization. Lack of previous hospitalizations may ‎indicate a fewer relapses over the history of the illness.‎

Treatment adherence is an important factor when it comes to bipolar disorder. Many studies ‎have shown that over 60% of the patients did not adhere to treatment. Non-adherence to ‎treatment and discontinuation of mood stabilizers increase the risk of relapse (11). The results ‎of this study is in agreement with previous studies because it showed that the risk of ‎recurrence was higher in patients who stopped drug consumption. In 2006, Altman et al., in a ‎systematic review, assessed the predictors of relapse in bipolar disorder (19). According to ‎their results, the factors associated with longer survival times included psychotherapy, social ‎support and medication adherence.‎

The risk of recurrence in patients with bipolar I disorder was higher than patients with bipolar ‎II disorder. This can be attributed to more hospitalized patients with bipolar disorder type I ‎than patients with bipolar disorder type II (20).‎

In this study, by comparing the participants with those patients with manic episodes, we ‎found that the patients with depressed episodes were at higher risk of recurrence; this has ‎been confirmed by previous studies (13). The highest risk of recurrence was observed among ‎those patients who had mixed episodes. ‎

In this study, it was revealed that family history of mood disorders had no association with ‎recurrence. Nevertheless, the risk of recurrence was higher in patients with the family history ‎of mood disorder compared to those without a family history of mood disorder. This can be ‎attributed to the decreased age of onset and increased severity of the disease. A higher ‎likelihood of hospitalization had been found in patients with family history of mood disorders ‎‎ (21).‎

Kessing et al used frailty models (22) to estimate the effect of the number of episodes on the ‎rate of recurrence taking into account the individual frailty toward recurrence. They ‎concluded the risk of subsequent recurrence increased with the number of episodes. ‎Degenhardt et al (23) used Cox Proportional Hazard models to determine the predictors of ‎relapse/recurrence in bipolar I disorder. In addition, de Dios et al. (15) found the predictors of ‎recurrence in bipolar disorders in Spain using logistic regression analysis. They concluded that ‎the factors significantly related to relapse were living setting and total number of previous ‎episodes. ‎

Medication is an essential component of treatment for patients with bipolar disorder; in ‎addition, psychosocial treatments and electroconvulsive therapy may further improve patients' ‎condition (24, 25). Although in this study patients who received both medication and ‎electroconvulsive therapy had a less risk of recurrence, those patients who received both ‎medication and psychosocial treatments (psychotherapy) had an increased risk of recurrence. ‎This may be due to the fact that most of the patients did not complete psychotherapy as it ‎lasts for a long time and is highly costly in Iran.

## Limitations

The limitations of this study were the absence of some data in patients' records and the ‎retrospective design. Despite these limitations, this study revealed some associated variables ‎with recurrence in the target population having an average income. The results may be useful ‎for physicians, psychiatrists and psychologists in preventing relapse.‎

## Conclusion

According to the results, several variables affect recurrence including occupation, type of ‎bipolar disorder according to DSM-IV-TR, and cessation of drugs use. Patients who received ‎both medication and electroconvulsive therapy showed a decreased risk of recurrence. ‎
